# Health and healthcare disparities among U.S. women and men at the intersection of sexual orientation and race/ethnicity: a nationally representative cross-sectional study

**DOI:** 10.1186/s12889-017-4937-9

**Published:** 2017-12-19

**Authors:** Mai-Han Trinh, Madina Agénor, S. Bryn Austin, Chandra L. Jackson

**Affiliations:** 1 Department of Mathematics and Statistics, University of Maryland Baltimore County, Baltimore, MD USA; 2 Department of Social and Behavioral Sciences, Harvard T.H. Chan School of Public Health, Boston, MA USA; 3 Division of Adolescent and Young Adult Medicine, Boston Children’s Hospital, Boston, MA USA; 4 Epidemiology Branch, National Institute of Environmental Health Sciences, National Institutes of Health, Department of Health and Human Services, 111 TW Alexander Drive, Research Triangle Park, NC 27709 USA

**Keywords:** Sexual orientation, Health behaviors, Health outcomes, Health Disparities

## Abstract

**Background:**

Research has shown that sexual minorities (SMs) (e.g. lesbian, gay, and bisexual individuals), compared to their heterosexual counterparts, may engage in riskier health behaviors, are at higher risk of some adverse health outcomes, and are more likely to experience reduced health care access and utilization. However, few studies have examined how the interplay between race and sexual orientation impacts a range of health measures in a nationally representative sample of the U.S. population.

**Methods:**

To address these gaps in the literature, we sought to investigate associations between sexual orientation identity and health/healthcare outcomes among U.S. women and men within and across racial/ethnic groups. Using 2013–2015 National Health Interview Survey data (*N* = 91,913) we employed Poisson regression with robust variance to directly estimate prevalence ratios (PR) comparing health and healthcare outcomes among SMs of color to heterosexuals of color and white heterosexuals, stratified by gender and adjusting for potential confounders.

**Results:**

The sample consisted of 52% women, with approximately 2% of each sex identifying as SMs. Compared to their heterosexual counterparts, white (PR = 1.25 [95% confidence interval (CI): 1.08–1.45]) and black (1.54 [1.07, 2.20]) SM women were more likely to report heavy drinking. Hispanic/Latino SM women and men were more likely to experience short sleep duration compared to white heterosexual women (1.33 [1.06, 1.66]) and men (1.51 [1.21, 1.90). Black SM women had a much higher prevalence of stroke compared to black heterosexual women (3.25 [1.63, 6.49]) and white heterosexual women (4.51 [2.16, 9.39]). White SM women were more likely than white heterosexual women to be obese (1.31 [1.15, 1.48]), report cancer (1.40 [1.07, 1.82]) and report stroke (1.91 [1.16, 3.15]. White (2.41 [2.24, 2.59]), black (1.40[1.20, 1.63]), and Hispanic/Latino SM (2.17 [1.98, 2.37]) men were more likely to have been tested for HIV than their heterosexual counterparts.

**Conclusions:**

Sexual minorities had a higher prevalence of some poor health behaviors, health outcomes, and healthcare access issues, and these disparities differed across racial groups. Further research is needed to investigate potential pathways, such as discrimination, in the social environment that may help explain the relationship between sexual orientation and health.

**Electronic supplementary material:**

The online version of this article (10.1186/s12889-017-4937-9) contains supplementary material, which is available to authorized users.

## Background

Previous studies have shown that sexual minorities (SM; e.g., lesbian, gay, and bisexual individuals) are more likely to engage in health risk behaviors, including smoking and heavy drinking [1–3], and are at higher risk of poor health outcomes, such as obesity and cardiovascular disease [4, 5], compared to their heterosexual peers. Additionally, SMs are more likely than heterosexuals to have lower levels of both health insurance and a regular source of healthcare [3, 6, 7]. In 2016, Jackson et al. examined the relationship between sexual orientation identity and health behaviors, outcomes, and healthcare access and utilization and found that lesbians had a higher prevalence of obesity and stroke and that SMs were more likely to delay seeking healthcare due to cost, despite no difference in insurance status [8].

In 2011, the Institute of Medicine identified gaps in knowledge and set recommendations that included intersectional lesbian, gay, bisexual, and transgender health research aimed to illuminate how sexual orientation and race/ethnicity simultaneously influence health and healthcare [9]. Rooted in black feminist thought, intersectionality recognizes that multiple, mutually constitutive dimensions of identity (e.g., race, gender, class, sexual orientation) and systems of oppression affect individuals’ lived experiences [10, 11]. Intersectionality provides an important theoretical framework for empirical researchers to consider how the interactions of multiple, individual-level social identities and structural-level social inequalities explain disparate health outcomes [10].

It is well-known that racial/ethnic minorities disproportionately suffer from poor health outcomes and a lack of access to healthcare services [12]. This knowledge, along with the aforementioned findings on SM health, underscore the need to investigate health disparities while addressing the intersecting identities of both sexual orientation and race/ethnicity. Prior research in this area has found, for example, that SM women of color had greater risks of lifetime substance use problems [13] and were more likely to be obese [14] than heterosexual women of color. SM men were less likely to be obese [13].

However, few intersectional studies have examined how sexual orientation impacts a range of health measures in a nationally representative sample of both U.S. women and men. Few have also assessed sexual orientation disparities both within (SM vs. heterosexual of the same race/ethnicities) and across (SM of color vs. white heterosexual) racial/ethnic groups, which is a major contribution of this study. To address this important gap in the literature, this study investigated sexual orientation identity disparities in health behaviors, health outcomes, and healthcare access and utilization indicators in the context of racial/ethnic diversity. Due to potential experiences with stigmatization and discrimination, we hypothesized that sexual and racial/ethnic minorities would report poorer health behaviors and health outcomes and a decreased use of healthcare services compared to white heterosexuals and compared to heterosexuals of the same race/ethnicity.

## Methods

### Study participants

This study analyzed pooled data from the 2013, 2014, and 2015 waves of the National Health Interview Survey (NHIS), a series of cross-sectional household surveys conducted by the National Center for Health Statistics. A detailed description of the study design and procedures has been published elsewhere [15]. Briefly, the NHIS used a multistage probability design to allow for nationally representative sampling of the non-institutionalized U.S. civilian population. Trained U.S. Census Bureau interviewers conducted the surveys using computer assisted personal interviewing and collected data on a broad range of sociodemographic characteristics and health indicators. The response rate for sample adults was 81% (range: 80–82%). Study participants included in this study were non-Hispanic white, non-Hispanic black/African American, and Hispanic or Latino (henceforth, white, black/African American, and Hispanic/Latino) adults aged 18 to 85+ years (mean age: 47 ± 0.14 years). Participants were excluded if they had missing data (< 3%) on sexual orientation identity or race/ethnicity. Written informed consent was obtained from all study participants.

### Measures

#### Sexual orientation identity

Regarding sexual orientation identity, participants were asked “Which of the following best represents how you think of yourself?” Response options included “gay” or “lesbian,” “straight, that is, not lesbian or gay,” “bisexual,” “something else,” and “I don’t know the answer.” Men were asked if they were “gay” or “straight, that is, not gay,” and women were asked if they were “lesbian or gay” or “straight, that is, not lesbian or gay.” Due to sample size constraints, participants identifying as “something else” or “I don’t know the answer” were not included in analysis. Participants identifying as gay, lesbian, or bisexual were also grouped into a single “sexual minority” category due to a limited sample size.

#### Health behaviors

Current smoking status and alcohol consumption were categorized as current, former, or never. Heavy drinking was defined as >2 drinks for men and >1 drink for women on 3–7 days per week in the past year. We also investigated participants who consumed ≥5 drinks on at least 2 days among men and women in 2013 and ≥5 drinks on at least 2 days among men and ≥4 drinks on at least 2 days among women for 2014. Leisure-time physical activity was classified as never/unable, low, or high. Participants reported how many hours they habitually slept per day, which was categorized as <7, 7–8, and >8 hours.

#### Health outcomes

Body mass index (BMI) was calculated using participants’ self-reported height and weight, with overweight classified as BMI ≥25 kg/m^2^ and obesity as BMI ≥30 kg/m^2^. Heart disease, hypertension, diabetes, cancer, or stroke was based on participants’ report of a health professional's diagnosis. Functional limitation was defined as having difficulties doing any of several specified activities because of a physical, mental, or emotional health problem other than pregnancy. At least one injury or poisoning episode serious enough to seek medical advice or treatment in the past 3 months was also measured. Participants responded to the question, “During the past 30 days, how often did you feel so sad that nothing could cheer you up?” with “none of the time,” “a little,” “some,” “most,” or “all the time.” As a measure of feeling depressed, participants responded to the question, “How often do you feel depressed?” with either the options of “daily,” “weekly,” “monthly,” “a few times a year,” or “never.”

#### Healthcare access and utilization indicators

Participants were asked if they had at least one place they usually went when they were sick or needed health advice. They also reported health insurance coverage or not, and if it was through Medicaid. Delayed medical care seeking, excluding dental care, because of worry about the cost during the past 12 months was measured. Those aged 64 years and under were asked if they ever received an HPV vaccine, and if they ever had an HIV test (excluding tests during blood donations). Women reported if they had a Pap smear or Pap test in the past 12 months, and women aged 30 years or older were asked if they had a mammogram in the past 12 months. Self-reported health status was categorized as excellent or very good, good, and fair or poor.

#### Race/ethnicity

As a potential modifier of associations between sexual orientation identity and health-related factors, participants self-identified their race/ethnicity upon being asked, “What race or races do you consider yourself to be? Please select 1 or more of these categories,” and “Do you consider yourself to be Hispanic or Latino?” Race/ethnicity was limited to White, Black, and Latino/Hispanic because other groups had an insufficient sample size.

#### Sociodemographic characteristics

Marital status was categorized as married (or living with a partner); divorced, separated, or widowed; and never married. Education was placed into four categories of < high school, high school (including general equivalency diploma), some college, and ≥ college-level education. Annual household income was dichotomized as <$35,000 and ≥$35,000, and poverty status was dichotomized based on the U.S. Census Bureau’s poverty threshold for total family or individual income. The 23 major occupation groups were grouped into professional/management, support services, and laborer occupations. Participants reported being US- or non-US-born, and region of residence included Northeast, Midwest, South, and West.

### Statistical analysis

We estimated the prevalence of sociodemographic characteristics, health behaviors, health outcomes, and healthcare outcomes in relation to sexual orientation identity and race/ethnicity (stratified by sex/gender) using the direct standardization method for age and the 2010 U.S. Census as the standard population.

Multivariable Poisson regression with robust error variance was used to estimate prevalence ratios (PR) and 95% confidence intervals (CI) for the association between sexual orientation identity and health behaviors, health outcomes, and healthcare outcomes stratified by race/ethnicity for U.S. women and men, adjusting for potential confounders. PRs were used to compare both SM to heterosexual individuals in the same racial/ethnic group and SM to white heterosexual individuals among both women and men. An initial analysis examined gays/lesbians and bisexual individuals separately, but these data are not shown due to low sample size. Covariates selected a priori as potential confounders included age, educational attainment, annual household income, occupational class, self-reported health status, region of residence, marital/cohabiting status, and immigrant status.

Sampling weights, based on the NHIS multistage design with stratification, clustering, and oversampling of certain subpopulations (i.e. black, Hispanic, Asian, and those aged ≥65 years), were used for all estimates. The “subpop” command was used for variance estimation with Taylor series linearization in Stata, version 14 (Stata Corporation, College Station, Texas, USA). A two-sided *p*-value <0.05 was considered statistically significant.

## Results

Among 91,913 participants, their mean age was 47 ± 0.14 years, 52% were women, and approximately 2% of each sex/gender identified as SM (Additional file 1: Table S1). Among men, 1.7% identified as gay and 0.4% identified as bisexual. Among women, 1.4% identified as gay/lesbian, and 0.8% identified as bisexual. White SMs generally had higher socioeconomic status than SMs of color. The information in the tables following can also be viewed as figures in Additional files 2 and 3.

### Health behaviors

Table 1 shows that white (PR = 1.33 [95% CI:1.14, 1.56), black (PR = 1.47 [95% CI: 1.07, 2.02]), and Hispanic/Latina (PR = 1.75 [95% CI: 1.12, 2.73]) SM women were more likely to be current smokers than heterosexual women of the same race/ethnicity. Compared to white heterosexual women, white SM women were more likely to report heavy drinking (PR = 1.25 [95% CI: 1.08,1.45]); black SM women were more likely than black heterosexual women to report heavy drinking (PR = 1.54 [95% CI: 1.07, 2.20]). White and Hispanic/Latina SM women were more likely to report sleeping >8 hours than heterosexual women of the same race/ethnicity. Table 2 shows that white SM men had a 46% higher prevalence of current smoking (PR = 1.46 [95% CI: 1.24, 1.73]) and were also more likely to be former smokers (PR = 1.27 [[95% CI: 1.09, 1.47]) than white heterosexual men. As well as being more likely to be current drinkers compared to heterosexual men of the same race/ethnicity (Table 2), white (PR = 1.14 [[95% CI: 1.10, 1.18]), black (PR = 1.14 [[95% CI: 1.01, 1.29]), and Hispanic/Latino (PR = 1.10 [95% CI: 1.00, 1.21]) SM men were more likely to be current alcohol drinkers than white heterosexual men (Table 4). Hispanic/Latino SM women and men were more likely to experience short sleep duration compared to their Hispanic/Latino heterosexual peers as well as white heterosexual women (PR = 1.33 [95% CI: 1.06, 1.66]) and men (PR = 1.51 [95% CI: 1.21, 1.90]).

**Table 1:** Fully-adjusted prevalence ratios for health behaviors, health outcomes, and healthcare access and utilization indicators for sexual minorities compared to heterosexuals across race/ethnicity among U.S. women, National Health Interview Survey, 2013–2015 (*n* = 50,854)

	White sexual minority (reference: heterosexual) PR (95% CI)	Black sexual minority (reference: heterosexual) PR (95% CI)	Latina/Hispanic sexual minority (reference: heterosexual) PR (95% CI)
Sample Size	*n* = 827	*n* = 222	*n* = 187
Health Behaviors			
Smoking status (ref: never)			
Current	**1.33 (1.14–1.56)**	**1.47 (1.07–2.02)**	**1.75 (1.12–2.73)**
Former	**1.62 (1.40–1.87)**	0.94 (0.48–1.85)	1.51 (0.86–2.67)
Alcohol consumption (ref: never)			
Current	1.06 (0.97–1.15)	1.12 (0.92–1.38)	1.07 (0.85–1.34)
Former	**1.39 (1.00–1.94)**	0.72 (0.20–2.54)	0.98 (0.47–2.03)
Heavy drinking	**1.25 (1.08–1.45)**	**1.54 (1.07–2.20)**	1.38 (0.84–2.25)
5+ drinks on at least 2 days	**1.23 (1.06–1.42)**	1.37 (0.98–1.90)	**1.55 (1.14–2.12)**
Leisure-time physical activity (ref: high)			
Low	0.97 (0.86–1.10)	0.94 (0.75–1.20)	0.98 (0.78–1.22)
Never/unable	0.86 (0.72–1.01)	0.88 (0.71–1.09)	0.88 (0.66–1.18)
Sleep duration (ref: 7, 8 hours)			
< 7 hours	1.04 (0.91–1.19)	1.14 (0.96–1.35)	**1.32 (1.07–1.63)**
> 8 h	**1.38 (1.03–1.87)**	0.99 (0.59–1.67)	**1.62 (1.05–2.51)**
Health Outcomes			
Overweight (yes)	**1.12 (1.04–1.22)**	1.06 (0.96–1.18)	1.04 (0.87–1.25)
Obesity prevalence (yes)	**1.31 (1.15–1.48)**	1.08 (0.89–1.31)	0.97 (0.72–1.30)
Hypertension (yes)	**1.18 (1.00–1.38)**	0.85 (0.65–1.12)	0.78 (0.45–1.36)
Diabetes (yes)	1.05 (0.69–1.61)	0.99 (0.56–1.75)	0.82 (0.39–1.74)
Cancer (yes)	**1.40 (1.07–1.82)**	0.44 (0.14–1.33)	1.42 (0.62–3.28)
Heart disease (yes)	1.04 (0.78–1.39)	1.26 (0.57–2.78)	0.57 (0.25–1.26)
Stroke (yes)	**1.91 (1.16–3.15)**	**3.25 (1.63–6.49)**	0.54 (0.10–2.93)
Functional limitation (yes)	**1.27 (1.13–1.42)**	1.22 (0.98–1.51)	**1.38 (1.10–1.73)**
Injury (3 months)	**1.74 (1.21–2.49)**	1.38 (0.60–3.16)	**3.92 (2.07–7.42)**
Sadness (≥ mostly in past 30 days)	**1.48 (1.01–2.16)**	**2.33 (1.39–3.91)**	1.76 (0.87–3.56)
Depressed (≥ weekly)	**1.54 (1.19–1.99)**	**3.02 (1.87–4.87)**	**2.28 (1.40–3.71)**
Healthcare Access and Utilization			
Health insurance (no)	1.08 (0.76–1.54)	1.13 (0.81–1.58)	0.87 (0.63–1.21)
Medicaid (yes)	0.81 (0.60–1.11)	**0.70 (0.51–0.95)**	0.73 (0.53–1.01)
Usual healthcare place (yes)	**0.89 (0.81–0.99)**	0.95 (0.88–1.03)	0.91 (0.82–1.01)
Delay in healthcare because of Costs (yes)	1.33 (0.96–1.84)	1.28 (0.90–1.80)	**1.75 (1.24–2.47)**
HPV vaccine (yes)	1.21 (0.90–1.62)	1.33 (0.98–1.79)	1.33 (0.97–1.83)
HIV test (yes)	1.13 (0.91–1.41)	1.10 (1.00–1.20)	1.05 (0.87–1.26)
Pap smear (yes)	0.94 (0.73–1.22)	1.01 (0.88–1.16)	0.86 (0.69–1.08)
Mammogram (yes)	0.67 (0.42–1.07)	1.15 (0.89–1.48)	**0.61 (0.37–0.99)**

The boldfaced entries that appear to be non-significant may be the ones with 1.00 as a confidence interval bound. These entries are boldfaced because all entries in the tables were rounded for conciseness, but without rounding, the boldfaced CIs were significant (e.g., CI:[1.004, 2])

**Table 2:** Fully adjusted prevalence ratios for health behaviors, health outcomes, and healthcare access and utilization indicators for sexual minorities compared to heterosexuals across race/ethnicity among U.S. men, National Health Interview Survey, 2013–2015 (*n* = 41,059)

	White sexual minority (reference: heterosexual) PR (95% CI)	Black sexual minority (reference: heterosexual) PR (95% CI)	Latino/Hispanic sexual minority (reference: heterosexual) PR (95% CI)
Sample Size	*n* = 759	*n* = 122	*n* = 187
Health Behaviors			
Smoking status (ref: never)			
Current	**1.46 (1.24–1.73)**	1.34 (0.80–2.23)	1.24 (0.81–1.88)
Former	**1.27 (1.09–1.47)**	0.68 (0.36–1.30)	1.09 (0.66–1.79)
Alcohol consumption (ref: never)			
Current	**1.14 (1.10–1.18)**	**1.21 (1.06–1.38)**	**1.13 (1.02–1.25)**
Former	**1.96 (1.39–2.78)**	1.05 (0.54–2.02)	0.79 (0.29–2.14)
Heavy drinking	1.11 (0.94–1.31)	1.52 (0.90–2.55)	1.14 (0.62–2.01)
5+ drinks on at least 2 days	1.03 (0.91–1.16)	1.20 (0.80–1.81)	1.08 (0.80–1.47)
Leisure-time physical activity (ref: high)			
Low	0.97 (0.85–1.10)	0.66 (0.42–1.02)	0.88 (0.65–1.19)
Never/unable	1.15 (0.98–1.36)	0.99 (0.67–1.47)	1.02 (0.76–1.37)
Sleep duration (ref: 7, 8 hours)			
< 7 h	1.10 (0.94–1.29)	0.83 (0.58–1.18)	**1.37 (1.10–1.71)**
> 8 h	1.16 (0.83–1.62)	1.00 (0.47–2.11)	0.99 (0.55–1.80)
Health Outcomes			
Overweight (yes)	**0.90 (0.83–0.98)**	0.84 (0.65–1.08)	**0.77 (0.64–0.93)**
Obesity prevalence (yes)	0.97 (0.82–1.15)	0.89 (0.58–1.36)	0.85 (0.60–1.20)
Hypertension (yes)	**1.19 (1.04–1.38)**	1.25 (0.87–1.80)	**1.71 (1.24–2.35)**
Diabetes (yes)	0.87 (0.61–1.24)	1.03 (0.55–1.95)	1.34 (0.72–2.50)
Cancer (yes)	**1.70 (1.27–2.28)**	1.78 (0.66–4.81)	0.89 (0.32–2.44)
Heart disease (yes)	1.27 (0.97–1.66)	1.63 (0.82–3.21)	0.80 (0.43–1.50)
Stroke (yes)	0.96 (0.50–1.84)	0.41 (0.09–1.82)	1.49 (0.53–4.16)
Functional limitation (yes)	**1.20 (1.05–1.37)**	1.28 (0.87–1.89)	**1.63 (1.14–2.33)**
Injury (3 months)	0.75 (0.48–1.18)	1.40 (0.44–4.52)	0.81 (0.32–2.07)
Sadness (≥ mostly in past 30 days)	1.51 (0.82–2.80)	**3.70 (1.68–8.13)**	1.84 (0.73–4.63)
Depressed (≥ weekly)	**1.68 (1.21–2.33)**	2.52 (0.93–6.83)	1.57 (0.85–2.93)
Healthcare Access and Utilization			
Health insurance (no)	1.01 (0.76–1.33)	**1.61 (1.18–2.19)**	0.96 (0.60–1.55)
Medicaid (yes)	0.81 (0.49–1.36)	**0.56 (0.33–0.98)**	0.73 (0.33–1.63)
Usual healthcare place (yes)	**1.07 (1.03–1.11)**	0.99 (0.84–1.16)	**1.18 (1.08–1.28)**
Delay in healthcare because of Costs (yes)	**1.42 (1.12–1.80)**	1.39 (0.82–2.34)	1.29 (0.79–2.08)
HPV vaccine (yes)	**2.33 (1.50–3.62)**	2.14 (0.95–4.81)	**2.36 (1.27–4.36)**
HIV test (yes)	**2.41 (2.24 - 2.59)**	**1.40 (1.20–1.63)**	**2.17 (1.98–2.37)**

The boldfaced entries that appear to be non-significant may be the ones with 1.00 as a confidence interval bound. These entries are boldfaced because all entries in the tables were rounded for conciseness, but without rounding, the boldfaced CIs were significant (e.g., CI:[1.004, 2])

### Health outcomes

Table 1 shows that, compared to white heterosexual women, white SM women were more likely to have obesity (PR = 1.31 [[95% CI: 1.15, 1.48]), hypertension (PR = 1.18 [95% CI: 1.00, 1.38]), cancer (PR = 1.40 [95% CI: 1.07, 1.82]), and stroke (PR = 1.91 [95% CI: 1.16, 3.15]). Black SM women were more likely to have a stroke than white heterosexual women (Table 3) and had over a three-fold higher prevalence of stroke (PR = 3.25 [95% CI: 1.63, 6.49]) compared to black heterosexual women (Table 1). White and Hispanic/Latina SM women were more likely to have a functional limitation and to have sustained an injury or poisoning in the past 3 months compared to heterosexual women of the same race/ethnicity. White (PR = 1.54 [95% CI: 1.19, 1.99]), black (PR = 3.02 [95% CI: 1.87, 4.87]), and Hispanic/Latina (PR = 2.28 [95% CI: 1.40, 3.71]) SM women were more likely to feel depressed weekly or more frequently compared to heterosexual women of the same race/ethnicity. Compared to white heterosexual men, white SM men had a higher prevalence of hypertension (PR = 1.19 [95% CI: 1.04, 1.38]), cancer (PR = 1.70 [95% CI: 1.27, 2.28]), a functional limitation (PR = 1.20 [95% CI: 1.05, 1.37]), and depressed feelings (PR = 1.68 [95% CI: 1.21, 2.33]) (Table 2). Although Hispanic/Latino SM men did not have a different prevalence of health outcomes compared to white heterosexual men, they were more likely to have hypertension (PR = 1.71 [95% CI: 1.24, 2.35]) and a functional limitation (PR = 1.63 [95% CI: 1.14, 2.33]) than Hispanic/Latino heterosexual men.

**Table 3:** Fully adjusted prevalence ratios for health behaviors, health outcomes, and healthcare access and utilization indicators for sexual minorities compared to white heterosexuals across race/ethnicity among U.S. women, National Health Interview Survey, 2013–2015 (*n* = 33,774)

	White sexual minority (reference: heterosexual white) PR (95% CI)	Black sexual minority (reference: heterosexual white) PR (95% CI)	Latina/Hispanic sexual minority (reference: heterosexual white) PR (95% CI)
Sample Size	*n* = 827	*n* = 222	*n* = 187
Health Behaviors			
Smoking status (ref: never)			
Current	**1.33 (1.14–1.56)**	0.85 (0.62–1.16)	**0.64 (0.43–0.94)**
Former	**1.62 (1.40–1.87)**	**0.48 (0.25–0.93)**	0.87 (0.54–1.41)
Alcohol consumption (ref: never)			
Current	1.06 (0.97–1.15)	1.04 (0.86–1.25)	0.88 (0.70–1.11)
Former	**1.39 (1.00–1.94)**	0.55 (0.16–1.83)	0.78 (0.33–1.83)
Heavy drinking	**1.25 (1.08–1.45)**	1.34 (0.96–1.88)	0.77 (0.49–1.23)
5+ drinks on at least 2 days	**1.23 (1.06–1.42)**	0.81 (0.59–1.11)	1.29 (0.95–1.75)
Leisure-time physical activity (ref: high)			
Low	0.97 (0.86–1.10)	1.01 (0.80–1.27)	1.09 (0.87–1.38)
Never/unable	0.86 (0.72–1.01)	1.13 (0.91–1.39)	1.01 (0.75–1.36)
Sleep duration (ref: 7, 8 hours)			
< 7 hours	1.04 (0.91–1.19)	**1.52 (1.27–1.83)**	**1.33 (1.06–1.66)**
> 8 h	**1.38 (1.03–1.87)**	1.06 (0.62–1.81)	1.32 (0.85–2.05)
Health Outcomes			
Overweight (yes)	**1.12 (1.04–1.22)**	**1.45 (1.30–1.61)**	**1.26 (1.03–1.56)**
Obesity prevalence (yes)	**1.31 (1.15–1.48)**	**1.63 (1.35–1.98)**	1.18 (0.85–1.64)
Hypertension (yes)	**1.18 (1.00–1.38)**	**1.38 (1.06–1.79)**	0.82 (0.48–1.40)
Diabetes (yes)	1.05 (0.69–1.61)	1.33 (0.74–2.39)	1.24 (0.60–2.56)
Cancer (yes)	**1.40 (1.07–1.82)**	**0.16 (0.05–0.49)**	0.86 (0.39–1.90)
Heart disease (yes)	1.04 (0.78–1.39)	0.91 (0.41–2.01)	**0.45 (0.22–0.92)**
Stroke (yes)	**1.91 (1.16–3.15)**	**4.51 (2.16–9.39)**	0.74 (0.18–3.00)
Functional limitation (yes)	**1.27 (1.13–1.42)**	1.18 (0.95–1.45)	1.23 (0.99–1.52)
Injury (3 months)	**1.74 (1.21–2.49)**	0.96 (0.44–2.11)	**2.88 (1.13–7.35)**
Sadness (≥ mostly in past 30 days)	**1.48 (1.01–2.16)**	**2.07 (1.24–3.44)**	1.37 (0.70–2.68)
Depressed (≥ weekly)	**1.54 (1.19–1.99)**	**1.56 (1.01–2.43)**	1.37 (0.77–2.41)
Healthcare Access and Utilization			
Health insurance (no)	1.08 (0.76–1.54)	1.14 (0.82–1.57)	1.08 (0.76–1.54)
Medicaid (yes)	0.81 (0.60–1.11)	1.07 (0.76–1.49)	0.81 (0.60–1.11)
Usual healthcare place (yes)	**0.89 (0.81–0.99)**	0.99 (0.91–1.07)	**0.89 (0.81–0.99)**
Delay in healthcare because of Costs (yes)	1.33 (0.96–1.84)	1.07 (0.75–1.52)	1.33 (0.96–1.84)
HPV vaccine (yes)	1.21 (0.90–1.62)	**1.43 (1.06–1.93)**	1.21 (0.90–1.62)
HIV test (yes)	1.13 (0.91–1.41)	**1.84 (1.65–2.06)**	1.13 (0.91–1.41)
Pap smear (yes)	0.94 (0.73–1.22)	**1.34 (1.17–1.54)**	0.94 (0.73–1.22)
Mammogram (yes)	0.67 (0.42–1.07)	**1.39 (1.10–1.76)**	0.67 (0.42–1.07)

The boldfaced entries that appear to be non-significant may be the ones with 1.00 as a confidence interval bound. These entries are boldfaced because all entries in the tables were rounded for conciseness, but without rounding, the boldfaced CIs were significant (e.g., CI:[1.004, 2])

### Healthcare access and utilization

Despite no differences in health insurance status among SM women of all races/ethnicities (Table 1), Hispanic/Latina SM women were less likely to have had a mammogram in the past year (PR = 0.61 [95% CI: 0.37, 0.99]) and more likely to delay healthcare due to costs (PR = 1.75 [95% CI: 1.24, 2.47]) than Hispanic/Latina heterosexual women. White SM women were less likely to have a usual place for care compared to white heterosexual women (PR = 0.89 [95% CI: 0.81, 0.99]). In contrast, white (PR = 1.07 [95% CI: 1.03, 1.11]) and Hispanic/Latino (PR = 1.10 [95% CI: 1.02, 1.19]) SM men were more likely to have a usual place for care compared to white heterosexual men (Table 4), as well as compared to heterosexual men of the same race/ethnicity (Table 2). Black SM men were more likely to be uninsured than black heterosexual men (PR = 1.61 [95% CI: 1.18, 2.19]) and white heterosexual men (PR = 1.83 [95% CI: 1.35, 2.46]). White SM men were 42% more likely than white heterosexual men to delay healthcare because of costs (PR = 1.42 [95% CI: 1.12, 1.80]). SM men of all races/ethnicities were more likely to have received an HIV test than heterosexual men within race/ethnicity and white heterosexual men.

**Table 4:** Fully adjusted prevalence ratios for health behaviors, health outcomes, and healthcare access and utilization indicators for sexual minorities compared to white heterosexuals across race/ethnicity among U.S. men, National Health Interview Survey, 2013–2015 (*n* = 28,460)

	White sexual minority (reference: heterosexual white) PR (95% CI)	Black sexual minority (reference: heterosexual white) PR (95% CI)	Latino/Hispanic sexual minority (reference: heterosexual white) PR (95% CI)
Sample Size	*n* = 759	*n* = 122	*n* = 187
Health Behaviors			
Smoking status (ref: never)			
Current	**1.46 (1.24–1.73)**	1.03 (0.61–1.74)	0.74 (0.49–1.10)
Former	**1.27 (1.09–1.47)**	**0.45 (0.24–0.84)**	0.81 (0.50–1.31)
Alcohol consumption (ref: never)			
Current	**1.14 (1.10–1.18)**	**1.14 (1.01–1.29)**	**1.10 (1.00–1.21)**
Former	**1.96 (1.39–2.78)**	1.16 (0.63–2.13)	0.95 (0.32–2.80)
Heavy drinking	1.11 (0.94–1.31)	1.21 (0.73–1.98)	0.65 (0.37–1.15)
5+ drinks on at least 2 days	1.03 (0.91–1.16)	0.89 (0.59–1.34)	0.91 (0.66–1.24)
Leisure-time physical activity (ref: high)			
Low	0.97 (0.85–1.10)	0.69 (0.45–1.07)	0.92 (0.67–1.26)
Never/unable	1.15 (0.98–1.36)	1.05 (0.71–1.57)	1.16 (0.85–1.59)
Sleep duration (ref: 7, 8 hours)			
< 7 h	1.10 (0.94–1.29)	1.21 (0.84–1.73)	**1.51 (1.21–1.90)**
> 8 h	1.16 (0.83–1.62)	0.90 (0.42–1.90)	1.00 (0.56–1.82)
Health Outcomes			
Overweight (yes)	**0.90 (0.83–0.98)**	0.89 (0.69–1.14)	0.85 (0.71–1.03)
Obesity prevalence (yes)	0.97 (0.82–1.15)	1.01 (0.67–1.53)	0.97 (0.68–1.37)
Hypertension (yes)	**1.19 (1.04–1.38)**	1.39 (0.97–2.00)	1.34 (0.95–1.88)
Diabetes (yes)	0.87 (0.61–1.24)	1.43 (0.74–2.74)	1.61 (0.86–2.99)
Cancer (yes)	**1.70 (1.27–2.28)**	1.07 (0.43–2.63)	0.52 (0.20–1.38)
Heart disease (yes)	1.27 (0.97–1.66)	1.52 (0.77–3.00)	0.67 (0.37–1.22)
Stroke (yes)	0.96 (0.50–1.84)	0.46 (0.10–2.08)	1.71 (0.67–4.41)
Functional limitation (yes)	**1.20 (1.05–1.37)**	1.08 (0.74–1.58)	1.21 (0.84–1.72)
Injury (3 months)	0.75 (0.48–1.18)	1.06 (0.33–3.43)	0.56 (0.23–1.38)
Sadness (≥ mostly in past 30 days)	1.51 (0.82–2.80)	**3.47 (1.52–7.95)**	2.24 (0.89–5.66)
Depressed (≥ weekly)	**1.68 (1.21–2.33)**	1.67 (0.70–4.00)	1.21 (0.68–2.14)
Healthcare Access and Utilization			
Health insurance (no)	1.01 (0.76–1.33)	**1.83 (1.35–2.46)**	1.34 (0.79–2.26)
Medicaid (yes)	0.81 (0.49–1.36)	0.88 (0.50–1.55)	0.91 (0.37–2.24)
Usual healthcare place (yes)	**1.07 (1.03–1.11)**	0.99 (0.84–1.16)	**1.10 (1.02–1.19)**
Delay in healthcare because of Costs (yes)	**1.42 (1.12–1.80)**	1.25 (0.74–2.13)	1.06 (0.65–1.73)
HPV vaccine (yes)	**2.33 (1.50–3.62)**	2.14 (0.92–4.94)	**2.32 (1.28–4.21)**
HIV test (yes)	**2.41 (2.24 - 2.59)**	**2.72 (2.31–3.21)**	**2.48 (2.26–2.72)**

The boldfaced entries that appear to be non-significant may be the ones with 1.00 as a confidence interval bound. These entries are boldfaced because all entries in the tables were rounded for conciseness, but without rounding, the boldfaced CIs were significant (e.g., CI:[1.004, 2])

## Discussion

Using nationally representative data, we identified sexual orientation identity disparities in health behaviors, health outcomes, and healthcare access and utilization indicators across and within racial/ethnic groups for both U.S. women and men. We found that white and black SM women were more likely to be heavy drinkers compared to heterosexual women from the same racial/ethnic backgrounds. SM men of all races/ethnicities were more likely to be current drinkers compared to white and same-race/ethnicity heterosexual men. SM women of all races/ethnicities were also more likely to be current smokers than heterosexual women of the same race/ethnicity, while this increased prevalence of current smoking was only found in white SM men. White SM women as well as white and Hispanic/Latino SM men, were more likely to have hypertension than their heterosexual peers of the same race/ethnicity. White SM women and men had a higher prevalence of reported cancer. Black SM women had over a three-fold higher prevalence of stroke compared to black heterosexual women, who already have a disproportionately high prevalence of stroke. Hispanic/Latina SM women had almost a four-fold higher prevalence of sustaining an injury or poisoning in the past 3 months compared to Hispanic/Latina heterosexual women.

Prior studies have shown that substance use was higher among SMs than heterosexuals [13]; however, there has been limited research on substance use disparities by sexual orientation both within and across racial/ethnic minority groups. Prior research has found that, in general, SM women of color are at higher risk of substance use than heterosexual women of color, while SM men of color have equivalent or lower risk than heterosexual men of color [13, 16, 17]. Our results regarding sleep disparities among Hispanic/Latino SMs are consistent with that of a previous study, which found that non-white SMs were more likely to report short sleep duration than white heterosexuals and white SMs [18]. As a potential explanation, research suggests an association between discrimination and short sleep [19].

Prior research has found that SMs were more likely to report having received an HIV test than their sex/gender-matched heterosexual peers [8]. Comparing SMs to their sex/gender and race/ethnicity-matched heterosexual peers, we observed a higher prevalence of HIV testing for SM men of all races/ethnicities but not for SM women. Results on healthcare access and utilization may also differ by age group, as indicated in other studies [8, 20], but our sample size did not allow for age stratification. Additionally, we found no differences in insurance coverage between SMs and heterosexuals except for black SM men being less likely to have health insurance. Despite a lower prevalence of insurance coverage, black SM men did not report differences in having a usual place of care, nor did black or Hispanic SM women compared to their heterosexual counterparts. It would be important to investigate where these participants access care as emergency care vs. primary care services likely lead to different health outcomes. In contrast to this finding, prior research has found that black and Hispanic SM women lacked a regular source of care compared to heterosexual peers, with black SM women also having a lower prevalence of insurance than heterosexual women [14]. Moreover, Agénor et al. found that odds of Pap test use for women with only female sexual partners may be lower among black women but did not differ among Hispanic/Latina women [21]. In this study, a different dimension of sexual orientation (i.e., sexual orientation identity vs. sex/gender of sexual partners) was used and the aggregation of SM identities may explain our conflicting findings. Sexual orientation and/or race/ethnicity based stigma and discrimination affect SMs’ attitudes towards healthcare and may contribute to healthcare access disparities [20, 22].

As a potential explanation informed by the multiple minority stress model, unique experiences of stigma, prejudice, and discrimination may lead to psychological distress and chronic physiological stress that has deleterious effects on SM mental and physical health [23–25]. Minority stress processes in SMs can involve external events (e.g. interpersonal and institutional discrimination); expectations of such events and vigilance associated with expectations; internalized homophobia; and coping by concealment of sexual orientation [25]. Guided by intersectionality theory [11], some research on SMs of color shows that individuals in this group could experience “double (or triple, in the case of SM women of color) jeopardy”: racism from the white LGB community, sexism, and heterosexism from their racial/ethnic communities [26, 27]. These social stressors can create unique vulnerabilities that also have a multiplicative effect on health parameters, and future research should measure pathways that generate unique risks/protections for SMs of color. One study using both additive and multiplicative approaches to intersectionality showed that black SM women experienced heightened exposure to discrimination and poorer mental health relative to white SM women and black SM men, groups with which they shared two (of three) marginalized statuses [23]. Other studies suggest that SMs of color develop resilience in response to experiences with racism prior to “coming out” and that this resilience protects against the effects heterosexism-related stressors [27–29]. Resources such as personal coping and group solidarity may counteract minority stress and help explain findings in which multiple marginalized statuses do not necessarily lead to poorer mental and physical health outcomes. Previous studies tout the consideration of both risk and protective factors in health disparities research as the minority stress model may be overly simplistic [30, 31].

There are several limitations of this study. First, we had access to one dimension of sexual orientation (i.e., sexual orientation identity), but others (e.g., romantic/sexual attraction and partner sex/gender) may differentially impact health and explain more variation in the sexual orientation and health relationship [32, 33]. Additionally, sexual orientation is not fixed but fluid, historically contingent, and culturally specific, although our data are cross-sectional [34]; therefore, longitudinal studies are needed [9]. These findings also do not apply to individuals who identified as other than lesbian, gay, or bisexual or who did not know. Similarly, the survey uses a restricted definition of gender, and findings from this study unfortunately cannot inform knowledge on health disparities for individuals whose identities fall outside of the gender binary (e.g., transgender-identified individuals). Second, those who identified as gay, lesbian, or bisexual were grouped into a single category as SMs due to sample size constraints. Third, all data were self-reported by participants; therefore, some results (e.g. BMI, income) may be subject to reporting bias. Fourth, data on social stressors (e.g. stigma, discrimination) were not available, although minority stress has been linked to suboptimal health and may mediate the sexual orientation identity and health relationships investigated.

Despite these limitations, this study has important strengths. Using nationally representative data, we summarized findings on a broad range of understudied topics, beyond the common health concern of HIV/AIDS [35], in SM health research. We analyzed data from all available survey years and could stratify analyses among sexual orientation identities by sex/gender and race/ethnicity. Through comparisons of SMs to white heterosexuals and heterosexuals of the same race/ethnicity, this study contributes to the literature a documentation of both multiplicative and additive intersectionality.

## Conclusions

Male and female SMs of color had a higher prevalence of some suboptimal health behaviors, health outcomes, and healthcare access and utilization indicators compared to both heterosexuals of the same race/ethnicity and white heterosexuals of the same sex/gender. Further research should investigate drivers of these sexual orientation identity disparities. Future data collection should oversample SMs of color so it is possible to reliably estimate racial/ethnic disparities among LGBTQ populations in hopes of providing more detailed analyses that investigate the impact of their unique experiences on health. These data can help inform tailored policies, clinical practice, and public health strategies to promote healthy behaviors among SMs and address the sociocultural factors (e.g., prejudice, discrimination) that contribute to current disparities in health and healthcare.

## Additional files

Additional file 1: Table S1. Age-standardized socio-demographic characteristics, health behaviors, health outcomes, and healthcare access and utilization indicators by sexual orientation identity among U.S. men and women, National Health Interview Survey, 2013–2015 (*N* = 91,913). (DOCX 20 kb)

Additional file 2: Figure S1. Adjusted prevalence ratios for health behaviors, health outcomes, and healthcare access and utilization indicators among sexual minority men compared to (a) white heterosexual men and (b) heterosexual men within race/ethnicity. (DOCX 198 kb)

Additional file 3: Figure S2. Adjusted prevalence ratios for health behaviors, health outcomes, and healthcare access and utilization indicators among sexual minority women compared to (a) white heterosexual women and (b) heterosexual women within race/ethnicity. (DOCX 204 kb)

## Abbreviations

BMI: Body mass index
CI: Confidence interval
NHIS: National Health Interview Survey
PR: Prevalence ratios
SM: Sexual minorities
