# Supplementary material for: Health and healthcare disparities among U.S. women and men at the intersection of sexual orientation and race/ethnicity: a nationally representative cross-sectional study
Source: BMC Public Health. 2017 Dec 19;17:964. doi: 10.1186/s12889-017-4937-9 (PMC5735619; doi:10.1186/s12889-017-4937-9)
Supplement: Supplementary file 1 — Age-standardized socio-demographic characteristics, health behaviors, health outcomes, and healthcare access and utilization indicators by sexual orientation identity among U.S. men and women, National Health Interview Survey, 2013–2015 (N = 91,913). (DOCX 20 kb) [file 12889_2017_4937_MOESM1_ESM.docx]

**Supplemental Figure 1. Adjusted prevalence ratios for health behaviors, health outcomes, and healthcare access and utilization indicators among sexual minority men compared to (a) white heterosexual men and (b) heterosexual men within race/ethnicity**

**a)**

**
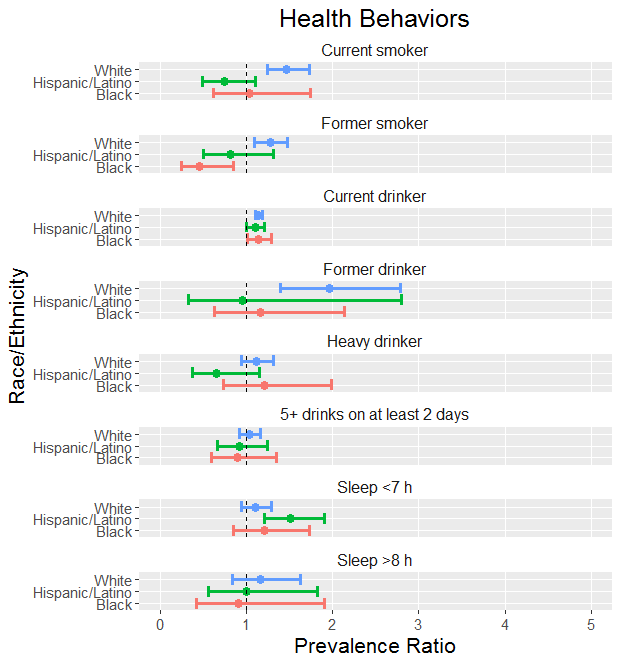

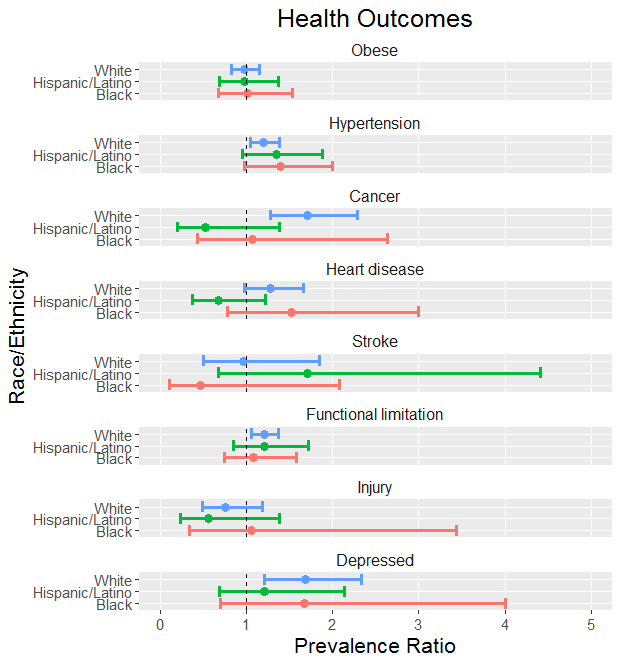

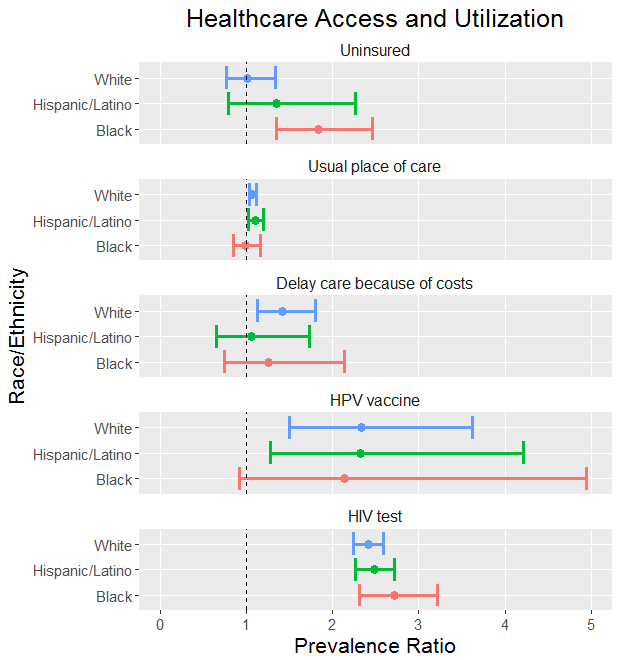
q**

**
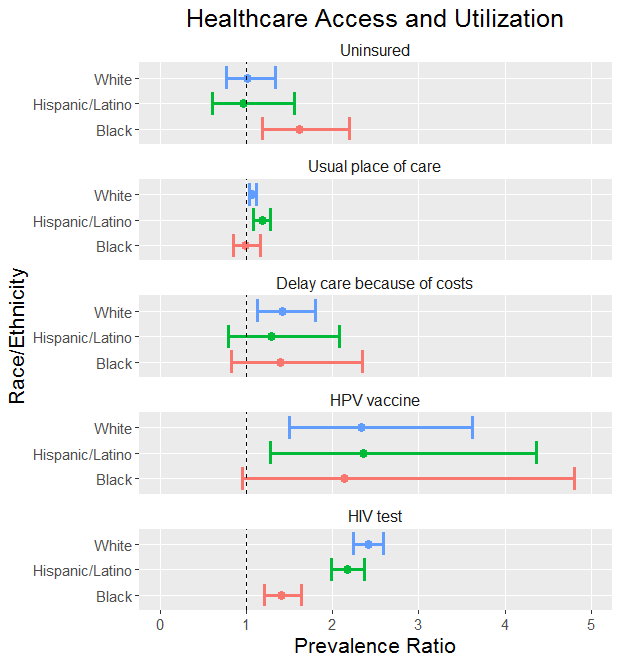

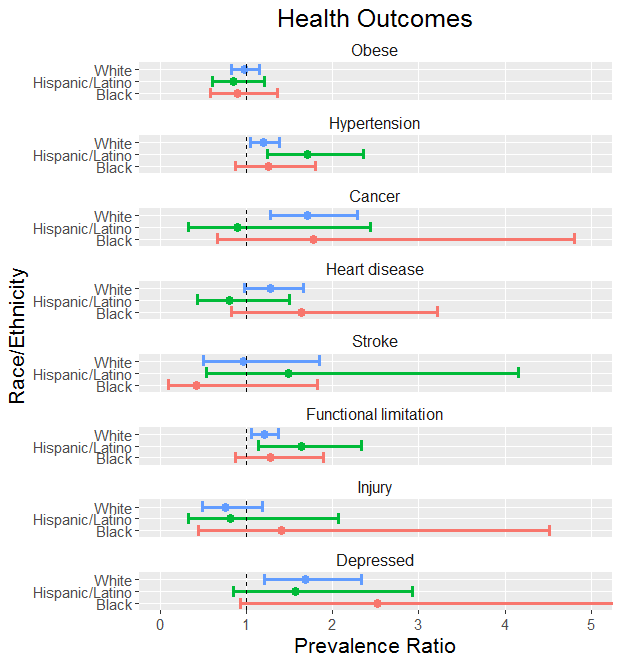

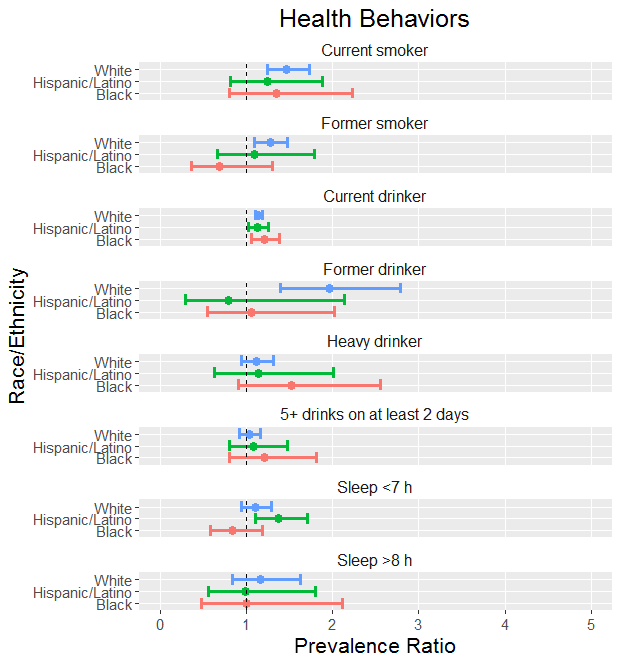
b)**
