# Supplementary material for: Health and healthcare disparities among U.S. women and men at the intersection of sexual orientation and race/ethnicity: a nationally representative cross-sectional study
Source: BMC Public Health. 2017 Dec 19;17:964. doi: 10.1186/s12889-017-4937-9 (PMC5735619; doi:10.1186/s12889-017-4937-9)
Supplement: Supplementary file 3 — Adjusted prevalence ratios for health behaviors, health outcomes, and healthcare access and utilization indicators among sexual minority women compared to (a) white heterosexual women and (b) heterosexual women within race/ethnicity. (DOCX 204 kb) [file 12889_2017_4937_MOESM3_ESM.docx]

**Supplemental Table. Age-standardized socio-demographic characteristics, health behaviors,**

**health outcomes, and healthcare access and utilization indicators by sexual orientation**

**identity among U.S. men and women, National Health Interview Survey, 2013-2015 (N = 91,913)**

|  | **Men (n = 41,059)** | | | | **Women (n = 50,854)** | | | |
| --- | --- | --- | --- | --- | --- | --- | --- | --- |
|  | **Heterosexual** | | **Sexual Minority** | | **Heterosexual** | | **Sexual Minority** | |
|  | **n** | **%** | **n** | **%** | **n** | **%** | **n** | **%** |
| **Sample Size** | 39,991 | 97.9 | 1,068 | 2.1 | 49,618 | 97.7 | 1,236 | 2.3 |
| **Sociodemographics** |  | | | | | | | |
| Race/ethnicity |  | | | | | | | |
| White | 27,392 | 73.6 | 759 | 78.9 | 32,538 | 72.5 | 827 | 76.0 |
| Black | 5,359 | 11.2 | 122 | 8.7 | 7,909 | 12.8 | 222 | 13.1 |
| Latino/Hispanic | 7,240 | 15.2 | 187 | 12.4 | 9,171 | 14.7 | 187 | 10.8 |
| Born in U.S. | 34,095 | 86.2 | 967 | 92.5 | 42,459 | 86.9 | 1,135 | 91.9 |
| Educational attainment |  | | | | | | | |
| < High school | 5,141 | 11.7 | 59 | 5.8 | 6,337 | 10.9 | 105 | 7.4 |
| High school graduate | 11,939 | 29.6 | 212 | 18.7 | 13,612 | 28.0 | 263 | 19.8 |
| Some college | 11,777 | 29.1 | 328 | 31.6 | 16,019 | 32.2 | 435 | 34.7 |
| ≥ College | 10,986 | 29.7 | 468 | 44.0 | 13,456 | 28.9 | 432 | 38.0 |
| Unemployed (yes) | 14,137 | 37.0 | 332 | 43.1 | 23,258 | 46.5 | 427 | 43.4 |
| Annual household income  (<$35,000 per year) | 13,929 | 28.3 | 409 | 32.6 | 20,475 | 34.0 | 583 | 35.8 |
| Living in poverty | 5,331 | 10.3 | 148 | 11.0 | 8,820 | 13.5 | 299 | 15.2 |
| Occupation |  | | | | | | | |
| Professional/management | 8,377 | 24.1 | 310 | 28.1 | 6,966 | 16.3 | 225 | 21.7 |
| Support services | 9,598 | 25.7 | 469 | 47.0 | 28,239 | 63.0 | 659 | 58.0 |
| Laborers | 19,874 | 50.2 | 260 | 24.9 | 10,207 | 20.7 | 280 | 20.3 |
| Marital status |  | | | | | | | |
| Married | 21,912 | 66.7 | 310 | 40.9 | 23,145 | 58.3 | 472 | 49.5 |
| Divorced/separated/widowed | 8,250 | 14.9 | 112 | 11.7 | 16,350 | 25.6 | 211 | 17.8 |
| Never married | 9,782 | 18.4 | 644 | 47.4 | 10,007 | 16.1 | 546 | 32.7 |
| Region of residence |  | | | | | | | |
| Northeast | 6,400 | 17.7 | 200 | 19.5 | 8,247 | 17.9 | 214 | 18.6 |
| Midwest | 8,860 | 23.7 | 169 | 16.6 | 10,530 | 23.0 | 234 | 22.5 |
| South | 14,245 | 37.4 | 388 | 35.2 | 18,670 | 38.8 | 452 | 36.8 |
| West | 10,486 | 21.2 | 311 | 28.7 | 12,171 | 20.3 | 336 | 22.1 |
| **Health Behaviors** |  | | | | | | | |
| Smoking status |  | | | | | | | |
| Never | 21,164 | 52.9 | 548 | 49.5 | 32,008 | 63.9 | 628 | 51.0 |
| Former | 10,936 | 29.2 | 251 | 28.3 | 10,028 | 21.4 | 283 | 31.1 |
| Current | 7,849 | 17.9 | 267 | 22.2 | 7,528 | 14.6 | 324 | 17.9 |
| Alcohol consumption |  | | | | | | | |
| Never | 1,829 | 13.7 | 23 | 5.6 | 4,189 | 24.5 | 53 | 15.3 |
| Current | 9,086 | 69.5 | 303 | 83.9 | 9,577 | 60.1 | 272 | 63.6 |
| Former | 2,106 | 16.7 | 32 | 10.5 | 2,514 | 15.4 | 46 | 21.2 |
| Heavy drinking | 7,192 | 21.4 | 251 | 27.8 | 7,579 | 21.2 | 295 | 26.9 |
| 5+ drinks on at least 2 days | 10,061 | 32.4 | 345 | 33.7 | 6,154 | 18.6 | 334 | 26.8 |
| Leisure-time physical activity |  | | | | | | | |
| Never/unable | 12,214 | 30.9 | 259 | 26.4 | 17,268 | 34.0 | 308 | 27.9 |
| Low/moderate | 12,980 | 33.3 | 338 | 31.8 | 15,472 | 32.0 | 408 | 32.9 |
| High | 14,665 | 35.7 | 470 | 41.8 | 16,762 | 34.0 | 513 | 39.2 |
| Sleep duration |  | | | | | | | |
| <7 hours | 12,369 | 30.5 | 324 | 28.5 | 15,634 | 31.1 | 449 | 32.2 |
| 7-8 hours | 24,388 | 61.3 | 649 | 60.9 | 29,379 | 59.5 | 659 | 55.5 |
| >8 hours | 3,234 | 8.2 | 95 | 10.6 | 4,605 | 9.4 | 128 | 12.3 |
| **Health Outcomes** |  | | | | | | | |
| Overweight prevalence | 28,468 | 73.2 | 635 | 61.4 | 28,866 | 60.2 | 760 | 64.5 |
| Obesity prevalence | 11,976 | 31.4 | 260 | 23.4 | 14,945 | 30.5 | 449 | 38.3 |
| Hypertension (yes) | 13,715 | 37.3 | 312 | 37.1 | 17,126 | 34.7 | 290 | 33.3 |
| Diabetes (yes) | 4,325 | 12.4 | 82 | 9.9 | 5,218 | 10.8 | 82 | 9.2 |
| Cancer (yes) | 3,608 | 10.5 | 95 | 13.5 | 5,189 | 11.1 | 108 | 13.1 |
| Heart disease (yes) | 4,911 | 13.7 | 117 | 14.4 | 5,452 | 11.0 | 98 | 10.8 |
| Stroke (yes) | 1,303 | 3.5 | 24 | 2.6 | 1,671 | 3.2 | 41 | 5.1 |
| Functional limitation (yes) | 13,193 | 34.5 | 331 | 36.4 | 21,314 | 43.6 | 539 | 48.5 |
| Injury (3 months) | 1,318 | 3.0 | 44 | 2.9 | 1,698 | 3.0 | 85 | 5.6 |
| Sadness (≥ mostly in past 30 days) | 1,075 | 2.6 | 39 | 3.1 | 1,933 | 3.7 | 83 | 5.6 |
| Depressed (≥ weekly) | 1,480 | 7.2 | 76 | 14.2 | 2,536 | 9.7 | 132 | 17.7 |
| **Healthcare Access and Utilization** |  | | | | | | | |
| Health insurance (no) | 6,303 | 13.0 | 156 | 12.2 | 6,022 | 10.8 | 189 | 10.4 |
| Medicaid (yes) | 3,274 | 7.2 | 84 | 7.6 | 7,014 | 11.3 | 215 | 12.6 |
| Usual healthcare place (yes) | 32,371 | 84.5 | 883 | 87.8 | 44,750 | 91.3 | 1,033 | 89.0 |
| Delay in healthcare because of  Costs (yes) | 3,831 | 7.8 | 162 | 10.9 | 5,542 | 10.1 | 247 | 15.0 |
| HPV vaccine (yes) | 763 | 2.3 | 57 | 5.7 | 3,750 | 9.1 | 215 | 14.8 |
| HIV test (yes) | 13,878 | 32.8 | 844 | 75.1 | 19,662 | 36.6 | 697 | 50.4 |
| Pap smear (past year) | - | - | - | - | 22,710 | 46.9 | 565 | 41.9 |
| Mammogram (past year) | - | - | - | - | 19,037 | 47.7 | 347 | 48.2 |
| **Self-reported general health status** |  | | | | | | | |
| Excellent/very good | 23,635 | 58.5 | 679 | 61.4 | 28,096 | 57.6 | 710 | 54.0 |
| Good | 10,655 | 27.1 | 251 | 24.2 | 13,869 | 27.6 | 320 | 29.0 |
| Fair/poor | 5,691 | 14.3 | 138 | 14.4 | 7,628 | 14.9 | 206 | 17.0 |
